# A Review of Petrol Burns in Australia and New Zealand

**DOI:** 10.1093/jbcr/irad008

**Published:** 2023-01-30

**Authors:** Nicholas Savage, Zakary Doherty, Yvonne Singer, Hana Menezes, Heather Cleland, Stephen J Goldie

**Affiliations:** Victorian Adult Burns Service, Alfred Hospital, 55 Commercial Road, Melbourne, Victoria, Australia; Victorian Adult Burns Service, Alfred Hospital, 55 Commercial Road, Melbourne, Victoria, Australia; Victorian Adult Burns Service, Alfred Hospital, 55 Commercial Road, Melbourne, Victoria, Australia; Victorian Adult Burns Service, Alfred Hospital, 55 Commercial Road, Melbourne, Victoria, Australia; Victorian Adult Burns Service, Alfred Hospital, 55 Commercial Road, Melbourne, Victoria, Australia; Department of Surgery, Central Clinical School, Monash University, Melbourne, Victoria, Australia; Victorian Adult Burns Service, Alfred Hospital, 55 Commercial Road, Melbourne, Victoria, Australia; Department of Surgery, Central Clinical School, Monash University, Melbourne, Victoria, Australia; College of Medicine and Public Health, Flinders University, Adelaide, South Australia, Australia

## Abstract

Petrol-related thermal burns cause significant morbidity and mortality worldwide and it has been established that they affect young males disproportionately. Beyond this, we sought to identify the difference in the characteristics and outcomes of burns between males and females in an international population. Such differences may highlight areas for future preventative strategies. The Burns Registry of Australia and New Zealand was used. Petrol burns that resulted in a hospital admission in those 16 years or older between January 2010 and December 2019 were included. A total of 2833 patients were included. The median age was 35 years with most patients being male (88%). Burns from a campfire or burnoffs were most common. Females were more likely to suffer burns due to assault or from deliberate self-harm. The total body surface area affected by burns was higher for females than males (10% vs 8%). Furthermore, females more frequently required ICU admission, escharotomies, and had a longer hospital length of stay. The unadjusted mortality rate for females was more than double the rate for males (5.8% vs 2.3%). This international study demonstrates that whilst men more frequently suffer petrol burns, women suffer more severe burns, require more intensive and longer hospitalizations and have a higher mortality rate. These findings may inform changes in preventative health policies globally to mitigate against these concerning findings.

Petrol-related thermal burns are a significant cause of morbidity, mortality, and cost in Australia and New Zealand as well as worldwide.^1–3^ Petrol, also called gasoline, in some countries, is used commonly in both domestic and industrial settings. Petrol poses risk for burn injury because it creates potentially explosive colorless vapor readily, requires a low minimum temperature for vapor ignition, has a high burning temperature, and is readily available. Injury is often associated with inappropriate use and handling techniques.^2^ Several studies have investigated the epidemiology of petrol burns in Australia and New Zealand finding that young males in rural and regional areas were the most commonly affected population.^1,4–8^ A previous study in the Australian state of Victoria found that petrol-related burn injury accounted for almost 20% of admissions to the state’s designated adult burn center.^1^ Young men were most at risk with alcohol and drug involvement being common. Furthermore, the mortality rate for patients with petrol burns in the Victorian study was 7.4%, which is more than 4-fold higher than the mortality rate of patients admitted to Australian and New Zealand Burn Centers with other types of burns.^9^ The majority of studies from the international literature show similar findings.^3,10–16^ To date, there have been no studies that have investigated gender-based differences associated with petrol burns. The aim of this study was to 1) describe the frequency, demographic profile, injury characteristics, inhospital outcomes, and cost for adults with petrol burns admitted to Australian and New Zealand burn centers; and 2) determine whether there is gender-based differences in the frequency, epidemiological characteristics and outcomes of adults who sustain petrol burns.

## METHODS

### Study Design

This was a retrospective observational cohort study. Ethics approval was obtained from the Alfred Health Human Research Ethics Committee (ref 583/20).

### Setting

This study was conducted in Australia and New Zealand where burn care is highly centralized. Patients who have sustained injuries that meet predefined criteria are transferred to 1 of 17 specialist Burn Services.^17^

### Data Source

Data were sourced from the Burns Registry of Australia and New Zealand (BRANZ). BRANZ is a bi-national clinical registry to which all Australian and New Zealand burns units report a minimum set of data for all patients admitted with burns for ≥24 hours, or less than 24 hours but required management of the burns in an operating theater or died patient within the first 24 hours of admission.

## PARTICIPANTS

### Inclusion Criteria

All patients, 16 years or older who were admitted to an Australian or New Zealand Burn Center between January 1, 2010 and December 31, 2019 (inclusive) were eligible for inclusion. Patients whose primary cause of burns were “*flames,*” in which “*accelerant involvement*” was confirmed, and the “accelerant type” was identified as “petrol,” as outlined in the BRANZ data dictionary were considered to have sustained petrol burns and were included in the study.^18^

### Data Management and Statistical Analysis

Data regarding demographic, injury severity, and inhospital outcome data for eligible cases were extracted. Comparisons were made between male and female patients.

Categorical variables were reported as frequencies with percentages, for numerical variables medians with 25% and 75% quartiles (IQR = Interquartile range) were reported (all data were not normally distributed). Data regarding the location the injury occurred were based on ARIA (Accessibility/Remoteness Index of Australia) levels as defined by the Australian Bureau of Statistics.^19^ Comparisons between male and female patients were made using the Wilcoxon rank sum test or Pearson’s chi-squared test where appropriate. A two-sided *P*-value <.05 was considered significant. The total annual cost of petrol burns admission was estimated using a method proposed by Ahn and Maitz.^15^ This cost analysis was only performed for patients that survived to hospital discharge as per the abovementioned method. All analysis was conducted using the R Project for Statistical Computing Software Version 1.3.9.

## RESULTS

There were 20,641 adult burns admissions registered during the study period in the BRANZ. Of these 2833 adults (13.7%) were admitted with petrol burns. Compared with non-petrol burns, patients who sustained petrol burns had higher mortality (2.7% vs 1.4%, *P* < .001), higher total body surface area (TBSA) (8% vs 3%, *P* < .001), and more frequent inhalation injury (13% vs 5%, *P* < .001).

### Patient and Injury Characteristics

**Figure 1.**
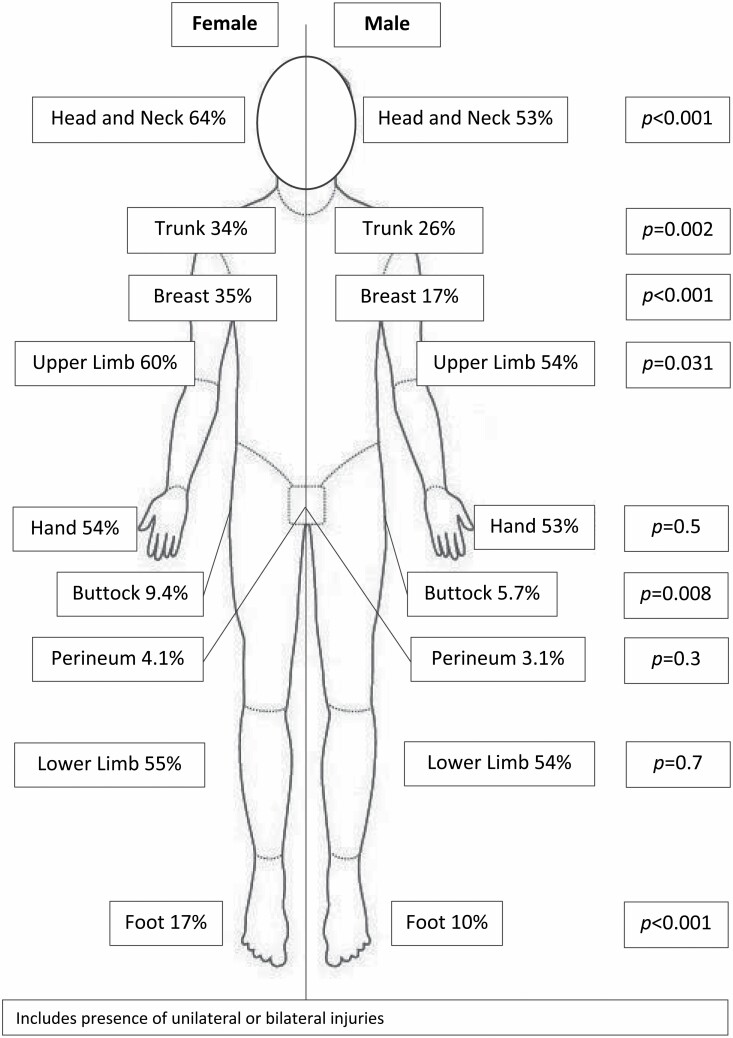
Anatomical locations of petrol burns.

Most patients were male (88%). The median age was 35 years (IQR; 24–48) with no significant differences between the ages of males and females were identified. The median size burn was 8% (4–15), full-thickness burns were sustained by 24% of patients, and 13% sustained inhalation injury. Overall women experienced more significant burns. Women had a higher median TBSA (%) than men (10% vs 8%) and more frequently had full-thickness burns and inhalation injury. Petrol burns most commonly affected the feet (89%), trunk (73%), and face (52%) (Figure 1).

### Circumstances Surrounding Petrol Injury

Petrol burns occurred more commonly on a weekend (21% per day for weekends vs 11% per day for weekdays). Burns were most commonly resulting from a campfire or burnoff, for both males and females. Burns were most commonly the result of campfires or burnoffs, with the mechanism more common in men. Lighters/matches were more common in women. The activity at the time of the burn varied between males and females: burns sustained whilst working for an income and during vehicle maintenance were both more common in men. Highly significant differences were seen with respect to the intent of the burn, both intentional self-harm (M:F = 6.9 vs 19%, *P* < .001) and assault (M:F = 2.4 vs 8.7%, *P* < .001) were more prevalent for females (Table 1). Thirty-one percent of injuries involved alcohol, drugs, or both; with alcohol being more common among males (25% vs 17%, *P* = .034). Place of burn injury and injury location were both different among genders (*P* = .009 and *P* = .003, respectively).

**Table 1.** Demographics and characteristics of petrol burns patients

Characteristic	Overall, n = 2833	Female, n = 348 (12.3%)	Male, n = 2485 (87.7%)	*P* ^2^
Age (median, IQR)	35 (24, 48)	36 (25, 48)	34 (24, 48)	.5
TBSA % (median, IQR)^g^	8.0 (4.0, 15.0)	10.0 (4.5, 23.2)	7.5 (4.0, 15.0)	<.001
Full-thickness burns present^h^	607 (24%)	103 (34%)	504 (23%)	<.001
Inhalation injury^i^	370 (13%)	72 (21%)	298 (12%)	<.001
Country				.003
Australia	2457 (87%)	284 (82%)	2173 (87%)	
New Zealand	376 (13%)	64 (18%)	312 (13%)	
Injury location^a^				.032
Major city	997 (44%)	112 (43%)	885 (45%)	
Inner regional	650 (29%)	72 (28%)	578 (29%)	
Outer regional	445 (20%)	51 (20%)	394 (20%)	
Remote	77 (3.4%)	7 (2.7%)	70 (3.5%)	
Very remote	73 (3.3%)	17 (6.6%)	56 (2.8%)	
Cause of burn^b^				<.001
Campfire/Burnoff	1309 (54%)	128 (44%)	1181 (55%)	
Vehicle engine/Parts	457 (19%)	52 (18%)	405 (19%)	
Other	295 (12%)	40 (14%)	255 (12%)	
Lighter/Matches	196 (8.1%)	49 (17%)	147 (6.9%)	
BBQ	91 (3.7%)	12 (4.1%)	79 (3.7%)	
Cigarette	83 (3.4%)	13 (4.4%)	70 (3.3%)	
Activity at time of burn^c^				<.001
Leisure	860 (44%)	97 (54%)	763 (43%)	
Vehicle maintenance	292 (15%)	11 (6.2%)	281 (16%)	
Gardening	289 (15%)	34 (19%)	255 (15%)	
Household maintenance	262 (14%)	26 (15%)	236 (13%)	
Working for income	232 (12%)	10 (5.6%)	222 (13%)	
Place of burn^d^				.009
Home	1555 (62%)	205 (65%)	1350 (61%)	
Recreation area	248 (9.8%)	31 (9.8%)	217 (9.8%)	
Other home (eg, friend’s home)	237 (9.4%)	21 (6.6%)	216 (9.8%)	
Farm	205 (8.1%)	16 (5.1%)	189 (8.5%)	
Public road	172 (6.8%)	33 (10%)	139 (6.3%)	
Workplace	111 (4.4%)	10 (3.2%)	101 (4.6%)	
Intent of burn^e^				<.001
Accidental	2461 (89%)	243 (73%)	2218 (91%)	
Intentional self-harm	232 (8.3%)	63 (19%)	169 (6.9%)	
Assault	87 (3.1%)	29 (8.7%)	58 (2.4%)	
Substance involvement^f^				.034
None	1618 (69%)	210 (76%)	1408 (68%)	
Alcohol only	572 (24%)	48 (17%)	524 (25%)	
Alcohol and drugs	84 (3.6%)	9 (3.3%)	75 (3.6%)	
Drugs only	80 (3.4%)	9 (3.3%)	71 (3.4%)	

^1^Wilcoxon rank sum test; Pearson’s chi-squared test.

Data missing for

^a^591 patients,

^b^402 patients,

^c^898 patients,

^d^305 patients,

^e^53 patients,

^f^479 patients,

^g^32 patients,

^h^314 patients, and

^i^18 patients.

### Treatment and Inhospital Outcomes

Overall, 4.9% of patients required an escharotomy, and 75% of patients underwent a burn wound management procedure in the operating room. During their hospital stay 26% of patients required admission to intensive care. Overall, 97.3% of patients survived to hospital discharge and the median hospital length of stay was 7.5 days (3.0–14.4). Patients who died had higher median TBSA (80% vs 7.5%, *P* < .001), more frequent full-thickness burn presence (89% vs 22%, *P* < .001) and inhalational burn presence (74% vs 11%, *P* < .001), and were more likely to die on a weekday (73% vs 58%, *P* < .005).

Women more frequently required escharotomies, and ICU admission during their hospital stay. The mortality rate for women with petrol burns was over double that of males (5.8% vs 2.3%), and the hospital LOS (Length of stay) of women was almost 3 days longer than men (Table 2). Intentional self-harm injuries resulted in larger TBSA burns, with more frequent full-thickness burn presence and higher mortality compared to accidental and assault injuries (Table 3). In assault injuries, women suffered larger TBSA (28% vs 12%, *P* = .016) and LOS (26 days vs 11 days, *P* = .006) with no difference in mortality (Table 4).

**Table 2.** Hospital treatment and outcome in petrol burns patients

Characteristic	Overall, N = 2833^1^	Female, N = 348^1^	Male, N = 2485^1^	*P* ^2^
Required theatre^a^	2049 (73%)	260 (75%)	1789 (73%)	.3
Required escharatomy^b^	55 (4.9%)	14 (11%)	41 (4.1%)	.001
Required ICU admission^c^	718 (26%)	129 (37%)	589 (24%)	<.001
Length of stay (days) (median, IQR)	7.5 (3.0, 14.4)	9.9 (4.3, 21.7)	7.1 (2.9, 13.7)	<.001
Survived to hospital discharge^d^	2732 (97.3%)	326 (94.2%)	2406 (97.7%)	<.001
Discharge location^e^				<.001
Home	2416 (86%)	256 (74%)	2160 (88%)	
Hospital transfer	127 (4.5%)	30 (8.7%)	97 (3.9%)	
Rehabilitation	95 (3.4%)	23 (6.6%)	72 (2.9%)	
Died	77 (2.7%)	20 (5.8%)	57 (2.3%)	
Other	94 (3.3%)	17 (4.9%)	77 (3.1%)	

^1^Median (IQR); n (%).

^2^Wilcoxon rank sum test; Pearson’s chi-squared test.

Data missing for

^a^24 patients,

^b^1711 patients,

^c^18 patients,

^d^24 patients, and

^e^24 patients.

**Table 3.** Petrol burn severity and outcomes

Characteristic	Overall, N = 2780	Accident, N = 2461	Intentional (self-harm), N = 232	Intentional (from other person), N = 87	*P* ^1^
TBSA (median, IQR)^a^	8.0 (4.0, 15.0)	7.0 (4.0, 13.0)	28.2 (10.0, 62.2)	15.0 (6.0, 28.5)	<.001
Full-thickness burn present^b^	583 (24%)	428 (20%)	125 (60%)	30 (39%)	<.001
Inhalational burn^c^	355 (13%)	210 (8.6%)	104 (45%)	41 (47%)	<.001
Length of stay (days) (median, IQR)	7.4 (3.0, 14.1)	6.9 (2.9, 12.7)	16.5 (4.8, 37.9)	13.5 (4.9, 29.6)	<.001
Survived to hospital discharge^d^	2,685 (97.4%)	2,437 (99.3%)	164 (75.9%)	84 (97.7%)	<.001

^1^Kruskal-Wallis rank sum test; Pearson’s chi-squared test; Fisher’s exact test.

Data missing for

^a^32patients,

^b^312 patients,

^c^17 patients, and

^d^23 patients.

**Table 4.** Causative intent of petrol burns and outcomes

Characteristic				*P* ^1^
Accident	Overall, N = 2461	Female, N = 243	Male, N = 2218	
TBSA (median, IQR)^a^	7, (4, 13)	8 (4, 15)	7 (4, 13)	.2
Inhalational burn^b^	210 (8.6%)	26 (11%)	184 (8.3%)	.2
Length of stay (days) (median, IQR)	7 (3, 13)	8 (4, 15)	7 (3, 13)	.002
Survived to hospital discharge^c^	2437 (99%)	240 (99%)	2197 (99%)	.7
Assault	Overall, N = 87	Female, N = 29	Male, N = 58	
TBSA (median, IQR)	15 (6, 28)	28 (8, 41)	12 (5, 24)	.016
Inhalational burn	41 (47%)	15 (52%)	26 (45%)	.5
Length of stay (days) (median, IQR)	14 (5, 30)	26 (12, 61)	11 (4, 20)	.006
Survived to hospital discharge^d^	84 (98%)	28 (97%)	56 (98%)	>.9
Intentional self-harm	Overall, N = 232	Female, N = 63	Male, N = 169	
TBSA (median, IQR)^e^	28 (10, 62)	25 (13, 59)	30 (10, 65)	>.9
Inhalational burn^f^	104 (45%)	27 (44%)	77 (46%)	.7
Length of stay (days) (median, IQR)	16 (5, 38)	26 (7, 49)	15 (3, 35)	.064
Survived to hospital discharge^g^	164 (76%)	46 (74%)	118 (77%)	.7

^1^Wilcoxon rank sum test; Fisher’s exact test; Pearson’s chi-squared test.

Data missing for

^a^28 patients,

^b^14 patients,

^c^6 patients,

^d^1 patient,

^e^4 patients,

^f^3 patients, and

^g^16patients.

Cost was estimated based on TBSA affected by burn. The median (IQR) cost was $93,344 ($56,888, $122,704) AUD per patient. The mean cost was $190733 AUD per patient or $540.3M AUD overall. This does not include the cost of transfer to hospital.

## DISCUSSION

This study provides a comprehensive review of adults who sustained petrol burns and were admitted to a burns unit across a bi-national population. Petrol burns represented 13.7% of admissions to burn centers in Australia and New Zealand with steady numbers of presentations across the time period. We identified that petrol burns affect genders disproportionately in terms of frequency, severity, and outcomes. While more men sustained petrol burn injuries than women, women sustained more severe burns and subsequently had worse outcomes than men. We found women had a higher rate of full-thickness burns, consistent with the findings of previous studies.^16^ Petrol burns resulted in worse injuries than non-petrol burns and were associated with high costs of treatment.

Comparing patterns of injury and outcomes between genders in petrol burns highlighted several differences. First, women and men had distinct causes, activities, and locations associated with injury. Second, there was a significant difference in substance involvement between genders. Third, the intent of injury was different between genders. Lastly, women suffered more severe burns and worse outcomes than men.

Consistent with other studies, we found campfire/burnoff to be the most common cause of injury.^1,7,20^ Place where injury occurred was significantly different between genders (*P* = .010). Females were more likely to be injured at home and public road while males were more likely to be injured at another home, farm, and workplace. Additionally, more than half of the injuries were outside the metropolitan area, increasing time for ambulance attendance, medical review, and burns unit transfer if required. Lastly, women had more severe injuries in terms of burn size, thickness, and presence of inhalation injury. Inhalation injury is a particularly important outcome as it has been found to be independently associated with mortality.^21^ However, we were not able to use regression analysis to explore this further due to the low sample size and event rate.

Our results suggest males are more likely to have substances involved with injury, particularly alcohol. Current literature suggests alcohol and drug use are common contributing factors to accelerant-related burns.^1,22,23^ These factors may influence the increased severity seen in petrol burns due to the reduced ability to move away from the source of thermal injury. Additionally, limiting petrol availability in social scenarios where alcohol or other drugs are used may reduce the occurrence of petrol burns.

Intent of injury was different among genders and outcomes were significantly different in assault injuries. Previous researchers have identified that intentional self-harm with petrol results in more severe burns by %TBSA and higher rates of mortality than nonintentional petrol burns.^5^ Current literature suggests that petrol burns are more frequently intentional compared to other types of burns.^17^ Males have been found to be more commonly affected by self-immolation in the West, while females have been found to be more commonly affected in the East.^18^ Mortality was higher in those suffering burns caused by intentional self-harm compared to accidental and assault injuries, a finding consistent with the literature.^1,23,24^ Our findings regarding increased TBSA burns and LOS in women suffering assault indicate the devastating nature of these injuries.

The overall mortality rate of 2.7% in our study was lower than the 7.4% found by Sreedharan et al. and 14.2% found by Barillo et al.^1,2^ These differences in findings are likely explained by differences in study inclusion criteria. Variations in Australian and New Zealand burn unit profiles, including the number and severity of burn injuries treated, identified by previous researchers can possibly explain the differences in mortality rates between our study and Sreedharan et al.’s study which analyzed data from one Australian burn center only. Furthermore, Barillo et al. included pediatric patients. Consistent with other studies, women suffered worse inhospital mortality.^19^ A possible explanation for this is differential immune responses following thermal injury between genders, particularly as it relates to the effect of estrogen.^20^ Estrogen appears to have a protective effect in non-burn trauma but not in burn injury.^21^ Others suggest that differences in mortality are related to skin thickness and body mass to muscle proportion.^22^ Another explanation is that women with less severe injuries are either not presenting or not being triaged for admission or burns unit management and therefore not being included in the BRANZ. Women have been shown to be less likely to be triaged to trauma centers despite similar injury severity and take longer to present following injury.^23^

Our findings represent potential targets for prevention that could reduce the burden of petrol burn injury. This is important given the substantial cost of petrol burns in Australia and New Zealand compared with other types of burns.^25^ It has been previously shown that costs of prevention may be offset by savings of reduced incidence and severity of burn injury.^26,27^ The typical petrol burn injury involves young males in the home environment undertaking leisure activities with the involvement of alcohol and other drugs. Education campaigns in high schools, increased signage at places of purchase of petrol, take away alcohol and camping equipment, at camping and recreational areas, (particularly in rural or regional areas), as well as warnings on petrol storage and transport receptacles regarding the safe use of petrol. Burns first aid may help reduce this type and burden of injury.^28^ This is particularly important for petrol burns, which commonly occur in regional and remote locations where first aid may be neglected or delayed.^29^ As in other thermal burns, the initial priority in first aid is to cool the injured area with running water, potentially salvaging already damaged tissue and preventing further injury.^30–32^

Women represent a vulnerable population for petrol burns; although they are less frequently injured than men, they suffered significantly worse injuries than men, and were more frequently injured due to self-harm or assault. Unfortunately, this is a global issue: women have been found to have higher mortality in a wide variety of populations including Nepal, India, Bangladesh, Iran, Vietnam, and the United States.^33–38^ With this in mind, the global burns community should engage with relevant women’s safety, prevention of violence, and/or self-harm stakeholder organizations to raise awareness of petrol-related violence and self-harm experienced by women around the world. Further research is also required to delineate the cause for differences between genders. However, facilitating early presentation may reduce the difference in outcomes between genders. Petrol burns being more common in regional/remote areas is likely related to the increased occurrence of campfires and burnoffs.^7,20^ The finding that people in regional/remote areas are disproportionately affected by petrol burns is significant because burn service provision in Australia and New Zealand revolves around large tertiary centers in metropolitan areas. Increased education for paramedics and regional/remote medical centers may increase referral patterns and improve outcomes.

Although our study captures a large population, it has some limitations. First, prehospital deaths are not captured by BRANZ, though they represent a significant portion of fatalities.^39^ BRANZ also does not capture people with burns not presenting to a burn center. Second, our analysis of injury event associated with petrol burns was limited by missing data.

## CONCLUSION

This international study of petrol burns across Australia and New Zealand confirms that the majority of patients involved are young men. However, self-harm and assault using petrol are more common in females. The injuries sustained by females are worse and their outcomes poorer. Our results should encourage reform regarding prevention strategies for education and community awareness, government policy, and legislation around the world.

## Notes

*Ethics approval and consent to participate*: Ethics approval was obtained from the Alfred Health Human Research Ethics Committee (ref 583/20).

*Funding*: No funding source applicable.

*Data availability*: Original data will be made available on request.
